# DEvis: an R package for aggregation and visualization of differential expression data

**DOI:** 10.1186/s12859-019-2702-z

**Published:** 2019-03-04

**Authors:** Adam Price, Adrian Caciula, Cheng Guo, Bohyun Lee, Juliet Morrison, Angela Rasmussen, W. Ian Lipkin, Komal Jain

**Affiliations:** Center for Infection and Immunity, Mailman School of Public Health of Columbia University, 722 West 168th St., New York, NY 10032 USA

**Keywords:** RNA-Seq, Transcriptomics, Differential expression, Visualization, Project Management, Data aggregation, Integration

## Abstract

**Background:**

Existing tools for the aggregation and visualization of differential expression data have discrete functionality and require that end-users rely on multiple software packages with complex dependencies or manually manipulate data for analysis and interpretation. Furthermore, at present, data aggregation and visualization are laborious, time consuming, and subject to human error. This is a serious limitation on the current state of differential transcriptomic analysis, which makes it necessary to expend extensive time and resources to reach the point where biological meaning can be interpreted. Such an approach for analysis also leads to scattered and non-standardized code, unsystematic project management and non-reproducible result sets.

**Results:**

Here, we present a differential expression analysis toolkit, DEvis, that provides a powerful, integrated solution for the analysis of differential expression data with a rapid turnaround time. DEvis has simple installation requirements and provides a convenient, user-friendly R package that addresses the issues inherent to complex multi-factor experiments, such as multiple contrast aggregation and integration, result sorting and selection, visualization, project management, and reproducibility. This tool increases the capabilities of differential expression analysis while reducing workload and the potential for manual error. Furthermore, it provides a much-needed encapsulation of scattered functionality, making large and complex analysis more efficient and reproducible.

**Conclusion:**

DEvis provides a wide range of powerful visualization, data aggregation, and project management tools that provide flexibility and speed in analysis. The functionality provided by DEVis increases efficiency of analysis and supplies researchers with new and relevant means for the analysis of large and complicated transcriptomic experiments. DEvis furthermore incorporates automatic project management capabilities, which standardizes analysis and ensures the reproducibility of results. After the establishment of statistical frameworks that identify differentially expressed genes, this package is the next logical step for differential transcriptomic analysis, establishing the critical framework necessary to manipulate, explore, and extract biologically relevant meaning from differential expression data.

## Background

RNA-sequencing (RNA-seq) has provided insights into mechanisms of host response to genetic and infectious diseases, host-pathogen interactions, cancer research, and plant biology [1–6]. Differential expression analysis of RNA-Seq data allows researchers to identify discriminating factors between experimental conditions. To that end, tools for performing differential expression analysis such as DESeq2, EdgeR, and Limma have been widely used in recent years [7–9]. While these tools provide a statistically rigorous framework for the identification of differentially-expressed genes, they require extensive effort on the part of bioinformaticians, who must coerce the resulting data from these packages into different data structures for interpretation or visualization. Furthermore, most experimental designs require complex contrasts that consider multiple factors and time points. This further complicates the analysis and makes identification of the most relevant genes with regard to biological factors or experimental conditions increasingly difficult as the scale of the experiment increases. These problems can be addressed by data aggregation methods that simplify combination and restructuring of transcriptomic data, and provide the flexible visualization tools needed to examine multiple factors of aggregated data, identify patterns of expression, and find transcriptomic profiles that correspond to biological and experimental conditions. Currently, however, few such tools are readily available, and researchers who want to aggregate or visualize their data are responsible for identifying and implementing methods that may be difficult to find, rely on obsolete dependencies, have complex setup procedures, or rely on external software packages that require the export and restructuring of data.

Here, we introduce DEvis, a comprehensive toolset for data aggregation, visual analytics, and exploratory analysis. DEvis addresses the limitations currently inherent to differential expression analysis by making it possible to manipulate and visualize transcriptomic data in easy and flexible ways. This toolset implements a wide range of visualizations for analyzing RNA-seq differential expression results, as well as data aggregation, transformation, sorting, and selection methods that smoothly integrate with the DESeq2 differential expression package, transforming DESeq2 into a more accessible platform for differential expression analysis with convenient visualization and data control facilities. Also, as DEvis, DESeq2, EdgeR and Limma all require similar data structures as input, most researchers can easily plug their input data into DEvis and quickly generate results. DEvis additionally implements a result management system that automatically organizes and stores analysis results, further simplifying the challenge of complex transcriptomic analysis, creation of publication-quality figures and contributing to the reproducibility of analysis.

The features and visualizations provided by DEvis are more extensive and varied than other packages for transcriptomic analysis. TRAPR includes plots for two-sample comparisons, heat maps, and filtration utilities, but is somewhat rigid in terms of layout, design, and customization of the data being displayed [10]. Another package, Scater, while providing visually appealing plots for summarization of data and dimensionality reduction, is primarily designed for single-cell RNA-Seq analysis and is limited in the types of data visualizations provided [11]. Glimma for RNA-Seq provides only dimensionality reduction and expression type visualizations [12]. Another package, PIVOT, offers similar categories of tools to DEvis, but differs in architecture, style, and usage [13]. For example, PIVOT uses a web-based user interface, which is visually appealing, but limits the potential for automation and pipeline integration that DEvis provides. A summary of the features provided by DEvis is provided in Table 1 and a general comparison of available features between visualization platforms is provided in Table 2.

**Table 1:** Summary of features implemented by DEvis

Feature Class	Available Methods
Data Preprocessing	TMM Normalization, rlog transform, Variance stabilization, Replicate merging, *P*-value / fold change cutoffs, Differential expression
Project Management	Standardized project output, Automated data / figure export, High resolution figure generation (pdf / png), Automatic figure size rescaling
Data Manipulation	Result set aggregation, Aggregation agreement density plots, P-value filtering, Fold change filtering, Metadata based subsetting, Metadata generation, Metadata modification, Gene ID/name transposition
Clustering	Euclidian-based distance, Poisson-based distance, Two-way hierarchical cluster heat mapping, Group-wise dendrograms
Dimensionality Reduction	MDS w/ confidence intervals, MDS w/ convex hulls, all genes / differentially expressed only
Distribution Plots	Pre-normalization / Post-normalization box plots, Group-wise fold change box plots, Group-wise fold change divergence plots
Expression Plots	Up/down regulation summary plots, volcano plots, gene heat maps, gene profile plots
Correlation Plots	Group-wise gene expression boxplot w/ Wilcoxon testing, Group-wise gene co-expression plots
Parameter Customization	Heat Map/ Profile plot data sorting (max/min/mean/variance/standard deviation), Custom legend/sample labeling, Metadata-based sample grouping, 6 layout/color scheme selections, Visualization data retrieval

**Table 2:** Comparison of features provided by several RNA-Seq data visualization platforms

	DEvis	PIVOT	TRAPR	scatr	Glimma	DESeq2
Summary Plots	✓	✓		✓		
Dimensionality Reduction Plots	✓	✓		✓	✓	✓
Cluster Plots	✓	✓				
Distribution Plots	✓	✓				✓
Expression / Significance Plots	✓	✓	✓	✓	✓	✓
Feature Statistics Plots	✓	✓				
Correlation / Co-expression Plots	✓	✓	✓			
Heat Maps	✓	✓	✓			
Volcano Plots	✓		✓		✓	
Data Aggregation	✓	✓				
Aggregation Plots	✓					
Normalization	✓	✓	✓	✓	✓	✓
Filtration / Feature Selection	✓	✓	✓		✓	
Project Management	✓	✓				
ID Conversion	✓	✓				
Publication Ready Figures	✓	✓		✓	✓	

## Implementation

### Data aggregation

Data aggregation is one of the core features of the DEvis package that makes it possible to examine and extract meaningful information from complex result sets. For example, analysis of time series data often requires the identification of differentially-expressed genes from each time point relative to a control sample, that may or may not also consist of time-controlled data. These types of experiments will produce a potentially dissimilar sets of differentially expressed genes for each time point contrast, meaning that direct comparisons between gene sets will not be immediately possible. DEvis makes it possible to combine results from multiple contrasts using union or intersection-based merging of data, which can be transformed and reshaped automatically to display different aspects of the data, such as sample similarity or group-wise expression changes, without relying on the user for data manipulation and reformatting. As a result, the researchers can efficiently investigate the similarities and differences from various combinations of the aggregated result sets and sensibly determine a master result set that can be filtered, subset, sorted, and visualized using other DEvis methods. For example, in a study with multiple time points for two experimental conditions, a researcher might be interested in identifying the differentially expressed genes unique to each condition regardless of the time point and then viewing changes in expression in both conditions for those genes across all time points. Researchers can visualize the levels of overlapping significant genes from aggregation of all time points for each condition and make informed decisions regarding the next steps. For instance, if there is little agreement in significant genes for a union-based aggregation of multiple time points for a single condition, a researcher could conclude that such a merging of data would likely introduce noise to the data set and consider an intersection-based approach or an aggregation of different combinations of result sets that would better characterize the condition under investigation. By providing easy and fast aggregation and an array of tools for generating and manipulating data, such as metadata generation, filtration, and subset functions, researchers have the ability to make educated decisions about their data analysis methods, the burden of data preparation and formatting is substantially reduced, and the potential for exploratory analysis is enhanced.

### Visualization

DEvis incorporates multiple methods of visualizations, each featuring easily configurable parameters that provide direct control over the data being visualized. DEvis also utilizes sample specific metadata that allows cross-group and multi-group data visualization based on user-defined parameters, making it possible to observe the effect of combining experimental factors on transcriptomic differences. Each visualization offers configurable layout and color schemes, with many providing filtering, sorting, and subsetting parameters for displaying and retrieving data, that facilitate exploratory analysis, and manipulation of data within the context of a complex experiment.

Visualizations for different aspects of transcriptomic analysis, such as batch effect identification, overall expression pattern investigation, sorting and filtration of significant differentially expressed genes, and co-expression analysis, are unique features of our tool. Prior to the investigation of differentially-expressed genes, the overall data set can be examined using hierarchical clustering, heat maps, MDS plots, and box plots. Clustering-based distance plots and dendrograms displaying metadata about each sample make it possible to quickly examine relationship of samples to one another and to identify outlying samples and batch effects. Additional plots, such as dispersion plots and boxplots, depicting expression metrics with regard to group-wise metadata can be used to identify large-scale changes in expression between conditions or to examine the effects of normalization. Individual genes can be examined using box plots based on group-wise metadata, making it possible to explore and identify the factors responsible for expression differences in genes of interest by splitting data by factors of interest, such as time point or experimental treatment conditions. Multi-dimensional scaling plots that incorporate convex hulls and confidence interval information further allow for identification of multi-factor causes as the source of large-scale differences between data.

**Fig. 1:**
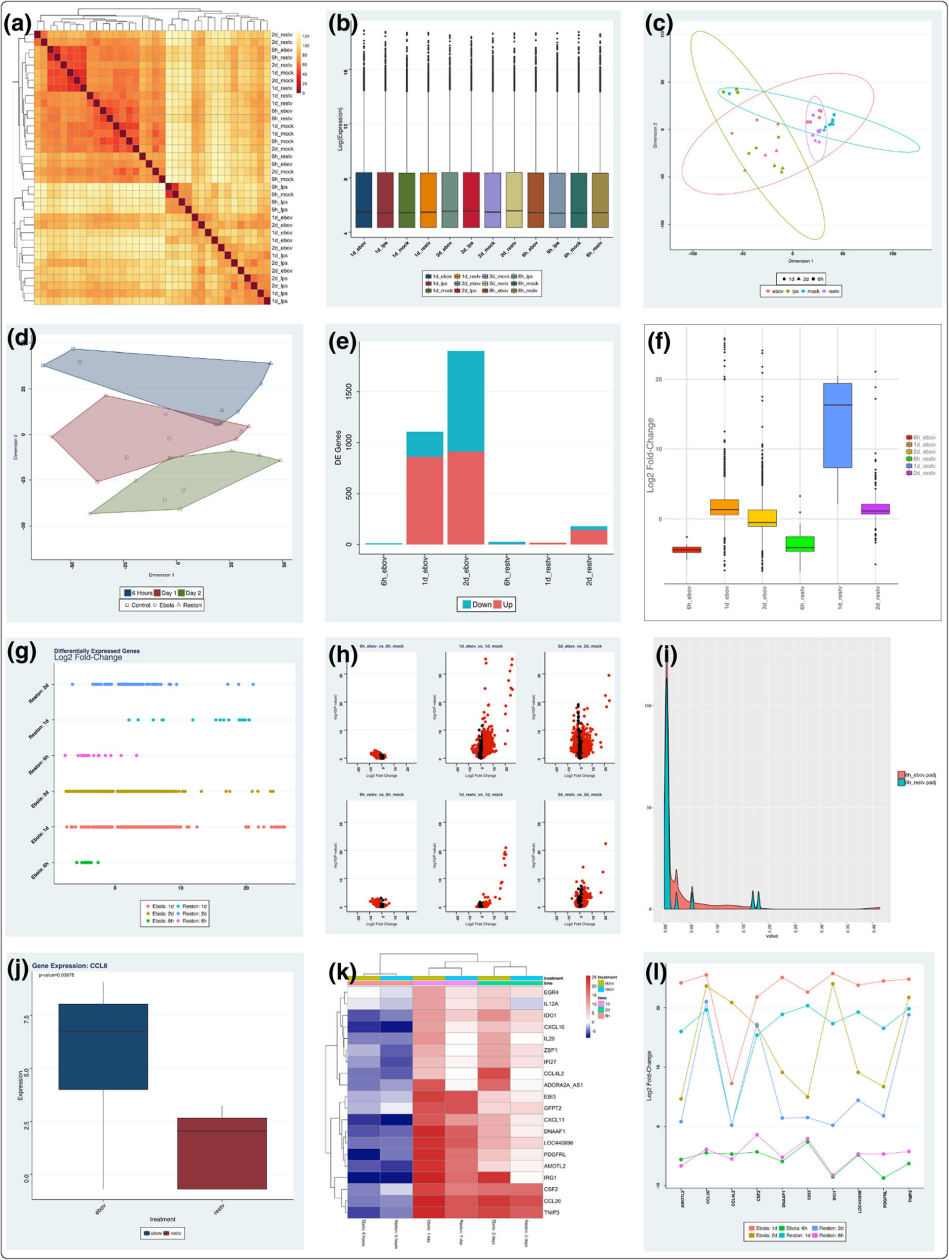
DEvis provides 16 visualizations such as sample distance plots (**a**), box plots for examining data normalization (**b**) MDS plots that show confidence intervals of clusters (**c**), MDS plots with convex hulls (**d**), summary count plots (**e**), box plots for examining fold change distribution (**f**), expression divergence plots (**g**), volcano plots (**h**), aggregation density plots (**i**), gene expression box plots with Wilcoxon testing (**j**), heat maps (**k**), and expression profile plots (**l**). Each visualization includes customizable sorting and selection parameters for data mining and exploratory analysis, as well as theme options for control of color schemes and layouts

After differential expression contrasts are performed and a master data set consisting of all comparisons of interest is aggregated, many additional visualizations become available. Density plots that display *p*-values or log fold-changes for the aggregated result set can be used to examine the impact of data aggregation, highlighting similarity or differences in each individual contrast with respect to the differentially-expressed genes identified in the aggregated master data set. Summary plots for displaying differentially-expressed gene counts and their respective expression levels for each contrast, as well as plots that show group-wise changes in differential gene expression and volcano plots are available. Heat maps and expression profile plots provide visualizations of gene expression changes across contrasts and provide sorting and filtering options that make it possible to identify the most important genes of interest with minimal effort. Finally, series plots allow genes to be clustered based on similarity of expression across multiple contrasts, making it possible to visualize and extract groups of genes based on co-expression across multiple contrasts. Examples of some of these plots can be seen in Fig. 1.

### Data organization

Several data organization functions are built into the DEvis package. A directory structure is created upon initialization, containing folders to house data files such as differentially-expressed gene lists, and visualized plots in either high resolution png or pdf format. Users can toggle whether data and plots should be automatically saved to the appropriate directory whenever a visualization is employed, standardizing project results and simplifying project management. This feature of DEvis simplifies the management of analysis results, standardizes the format and structure of results, and reduces the chance of human error, ensuring future reproducibility and providing a standard for result storage that can be easily navigated and understood long after completion of analysis.

## Results

An example analysis was performed, to validate the results and assess the features of DEvis, from a recently published RNA-Seq study on the pathogenicity of Ebola and Reston viruses in humans [4]. An in-depth version of this analysis is provided in the DEvis vignette document. For the purposes of this paper, however, a simplified explanation is provided as an overview of a straightforward analysis.

Data for time (6 h, 1d, 2d) and treatment (ebov, restv, lps, mock) in human macrophages was downloaded from SRA (SRP078152). Data were analyzed using DEvis and several preprocessing steps were performed before TMM normalization. Differential expression calculations were carried out followed by union-based aggregation of results. The results of merging for each time/virus combination were examined using gene significance density plots that showed strong agreement between significant genes at each time point between restv and ebov, and a subset of genes being significant in either restv or ebov, but not both (Fig. 1i). This was determined to be a valid aggregation for this data set, as these are the same family of viruses and the hypothesis of the original study was that restv was not pathogenic in humans, in which case this level of similarity would be expected. In parallel, two-way hierarchical clustering was performed to examine sample similarity (Fig. 1a), and pre-normalization (not shown) and post-normalization (Fig. 1b) plots were generated to check the integrity of the data, the effects of normalization, and to examine summaries of identified differentially expressed genes (Fig. 1e). Multi-dimensional scaling (MDS) with confidence intervals (Fig. 1c) and MDS with convex hulls (Fig. 1d) were used to analyze data clustering, based on differences in metadata, to identify the factors influencing the sample set as a whole and the subsets of differentially expressed genes. Distributions of expression levels were examined using boxplots (Fig. 1f) and dispersion plots (Fig. 1g), and significance and expression changes were visualized for all contrasts using volcano plots (Fig. 1h). Heat maps were used to investigate the expression of genes across contrasts using several sorting and selection criteria (Fig. 1k) and profiles of the expression changes in subsets of these genes were examined as a function of infection and time (Fig. 1l). Certain genes of interest, as identified in heat map analysis, were examined individually with regard to infection conditions using gene specific boxplots with Wilcoxon tests (Fig. 1j). Co-expression analysis was performed (not shown) and groups of differentially expressed genes with differences in patterns of expression over time were identified for each infection condition. Figures and relevant data in all steps were automatically organized and stored as image and data files.

In this example, each plot can be generated using a single line of code (as shown in the vignette), and exploratory analysis can be performed through the use of different parameters and metadata combinations for each visualization. If a bioinformatician were to perform the same types of analyses as shown here, substantial computations, data selection, and reformatting would be required, consuming time, increasing the potential for human error, and potentially reducing reproducibility.

## Conclusion

DEvis provides a user friendly and versatile tool set for aggregation, visualization, and exploratory analysis of transcriptomic data. This package provides a single repository of tools required for the analysis and investigation of complex transcriptomic data sets, as well as automated project management functionality that standardizes analysis, reduces workload, and creates reproducible results. Furthermore, it obliviates the need for maintaining multiple scripts and implementations of various software packages and their dependencies.

Extensive documentation is available for DEvis in the form of a technical manual that outlines the detailed functionality and parameter usage of each method, and a vignette document, which functions as a case-study and tutorial on the usage and caveats of DEvis. This vignette was created based on analysis of data from the previously outlined study on the pathogenicity of Ebola and Reston viruses in humans [4]. The technical documentation and vignettes are available with the DEVis package at https://github.com/price0416/DEvis.

## Availability and requirements

**Project name:** DEvis.

**Project home page:** https://github.com/price0416/DEvis

**Operating system(s):** Platform independent.

**License:** LGPL.

**Programming language:** R.

**Other requirements:** None.

**Any restrictions to use by non-academics:** LGPL license, open source.
